# Characterization of an Atypical Metalloproteinase Inhibitors Like Protein (Sbp8-1) From Scallop Byssus

**DOI:** 10.3389/fphys.2018.00597

**Published:** 2018-05-23

**Authors:** Xiaokang Zhang, Xiaoting Dai, Lulu Wang, Yan Miao, Pingping Xu, Pengyu Liang, Bo Dong, Zhenmin Bao, Shi Wang, Qianqian Lyu, Weizhi Liu

**Affiliations:** ^1^MOE Key Laboratory of Marine Genetics and Breeding, College of Marine Life Sciences, Ocean University of China, Qingdao, China; ^2^Laboratory for Marine Biology and Biotechnology, Qingdao National Laboratory for Marine Science and Technology, Ocean University of China, Qingdao, China; ^3^Laboratory for Marine Fisheries Science and Food Production Processes, Qingdao National Laboratory for Marine Science and Technology, Qingdao, China

**Keywords:** scallop, byssus, TIMP, bioadhesion, cross-linker, Cysteine

## Abstract

Adhesion is a vital physiological process for many marine molluscs, including the mussel and scallop, and therefore it is important to characterize the proteins involved in these adhesives. Although several mussel byssal proteins were identified and characterized, the study for scallop byssal proteins remains scarce. Our previous study identified two foot-specific proteins (Sbp7, Sbp8-1), which were annotated as the tissue inhibitors of metalloproteinases (TIMPs). Evolutionary analysis suggests that the *TIMP* genes of *Chlamys farreri* had gone through multiple gene duplications during evolution, and their potential functional roles in foot may have an ancient evolutionary origin. Focusing on the Sbp8-1, the sequence alignment and biochemical analyses suggest that Sbp8-1 is an atypical TIMP. One significant feature is the presence of two extra free Cys residues at its C-terminus, which causes the Sbp8-1 polymerization. Considering the fact that the no inhibitory activity was observed and it is mainly distributed in byssal thread and plaque, we proposed that this atypical Sbp8-1 may play as the cross-linker in scallop byssus. This study facilitates not only the understanding of scallop byssus assembly, also provides the inspiration of water-resistant materials design.

## Introduction

It is already well-known that adhesion is one of the most important physiological processes for marine molluscs, which is vital for food procurement, locomotion, defense, breeding and attachment (Gorb, 2008). In the marine environment, many organisms were adversely affected by tides and waves, and in response to this situation many marine molluscs have evolved their ability to live by adhering themselves to other materials (Yang et al., 2013). Like mussels and oysters can secrete adhesives and cement to affix themselves to the substratum (Wilker, 2011). Adhesive locomotion is an important strategy for gastropods, the mucus released from their foot play an important role in locomotion (Iwamoto et al., 2014). The tentacles in *Nautilus* can produce glue in a specialized glandular structure to pick up food or attach to substrate for mating (von Byern et al., 2012). Therefore, adhesion widely exists in marine molluscs indicating this is a crucial physiological event for these organisms. In addition, it is obvious that the marine adhesion material exhibits remarkable adhesion ability with water-resistance properties, providing inspiration for the design of new materials to meet diverse application requirements (Aldred, 2013). Therefore, extensive amounts of efforts were put to characterize the adhesives from sessile organisms such as mussels, barnacles, or tube-dwelling worms (Naldrett and Kaplan, 1997; Taylor and Waite, 1997).

So far, several significant features for marine mollusc adhesives were discovered based on the characterized mollusc adhesives, including the mussels and barnacles. First, Presence of post-translational modifications (PTM): Several different types of PTM are detected for the studied marine bioadhesive proteins. For example, phosphorylated proteins (Zhao et al., 2005) and hydroxylated DOPA (Wang and Stewart, 2013) were found in the cement of sandcastle worm and *P. californica* respectively. Glycosylation was detected in byssal threads of marine mussels (Sun et al., 2002), and the tube feet disk nectin presented phosphorylation and glycosylation (Toubarro et al., 2016). Second, Metal ions are found to be critical for the marine adhesive materials. Elemental analysis of cured *P. californica* glue revealed relatively large quantities of Ca and Mg, which are complexed with the peptidyl-phosphates in the heterogeneous sub granules (Wang and Stewart, 2013). And Fe^3+^-DOPA coordinative complexes were detected in the protective cuticle and bulk adhesive plaque of the byssus (Harrington et al., 2010). Third, so far, the identified marine adhesion proteins are non-conserved. For example, the protein compositions between scallop and mussel are significantly different, although they all belong to bivalves and adopt the byssus to attach to the substratum (Miao et al., 2015). Therefore, it is important to dissect and characterize the individual compositions of scallop byssus, which was important for the understanding its adhesive mechanisms.

Scallops produce and secrete specialized adhesives that work synergistically in water allowing them to attach themselves in marine environments. The secreted adhesive proteins solidified in sea water and shaped into byssus with excellent flexibility and toughness. In previous studies, we discovered 75 proteins from *Chlamys farreri* byssus based on transcriptomic approach and further identified seven foot-specific scallop byssal protein (Sbp) components based on proteomic approach (Miao et al., 2015). By sequence alignment, two proteins (Sbp7, Sbp8-1) were annotated as the tissue inhibitors of metalloproteinases (TIMPs) family. It is known that TIMPs can regulate important physiological activities by inhibiting metalloproteinases activity, which are proteinases that participate in extracellular matrix (ECM) degradation (Nagase and Woessner, 1999). In addition, it is suggested that TIMPs have other biological functions. For example, TIMPs are involved in erythroid-potentiating (Stetler-Stevenson et al., 1992) and cell growth–promoting (Hayakawa et al., 1992), and it is found that TIMPs can induce apoptosis and stimulate angiogenesis (Qi and Anand-Apte, 2015). TIMP also might be involved in antibacterial immune in mollusc (*Tegillarca granosa*) (Wang et al., 2012). Serine proteases and chorionic proteinase inhibitor from *Amphibalanus amphitrite* was identified, which is predicted to be possibly involved in either regulating the shell formation or functioning in immunity (Zhang et al., 2015). However, the knowledge for the discovered TIMP homologous (Sbp7, Sbp8-1) is insufficient, which precludes the understanding of its physiological function. Moreover, because TIMPs are present as multiple gene copies in the scallop genome, figuring out their evolutionary relationships may promote better understanding of expressional difference between different copies and provide insights into their potential functional divergence. Here our evolutionary analysis suggests that the TIMPs of *C. farreri* had gone through multiple gene duplications during evolution. Focusing on the Sbp8-1, which was mainly distributed in the byssal thread and plaque, detailed sequence alignment suggests that Sbp8-1 is an atypical TIMP with two extra free Cys residues at its C-terminus. More importantly, biochemical analysis in combination with mutagenesis suggests that two extra free Cys residues cause the Sbp8-1 polymerization. Finally considering the fact that the no inhibitory activity was observed, this atypical Sbp8-1 was proposed to function as the cross-linker in scallop byssus. This study will facilitate the biological function understanding of TIMP homologous (Sbp8-1) in byssus.

## Materials and Methods

### Sample Collection

The adult *Chlamys farreri* used in this study were purchased from the market in Qingdao and the scallops were kept in the aquarium with circulating sea water at around 19°C. Frosted glass was laid on the bottom of the aquarium in order that the scallops can secret adhesives to form byssus. All experiments in this study were repeated at least three times.

### Identification and Evolutionary Analysis of *TIMP* Genes

Putative *TIMP* genes in scallops and other molluscs were identified by performing blastp with an *e*-value threshold of 1e^-5^, using known *TIMP* genes of human and fly retrieved from NCBI protein database as queries. The conserved domain was identified by using the CDD tool^1^. And only the candidate genes containing NTR domain (Form complexes with metalloproteinases, such as collagenases, and irreversibly inactivate them) were kept for the following analysis. The identified TIMP protein sequences were aligned using clustalw in the MEGA 6.0 software (Tamura et al., 2013) and a phylogenetic tree of *TIMP* genes was constructed with the program MrBayes (ver3.2.6) (Huelsenbeck et al., 2001). The MCMC model-jumping method was performed to run for 1,000,000 generations with a sample frequency of 1000. In total, 1000 trees were produced, of which the first 250 were discarded as burn-in, while the remaining were summarized to get a consensus tree.

### Expression Analysis

The expression levels of *TIMP* genes were retrieved from the RNA-seq datasets of *C. farreri* that were generated from a whole-genome sequencing project (Li et al., 2017), including eleven adult tissues/organs (adductor muscle, smooth muscle, foot, hepatopancreas, blood, kidney, female gonad, male gonad, gill, eye, and mantle) and three different foot regions (tip region, middle region, and root region). Illumina sequencing reads were mapped to the genome of *C. farreri* by using the STAR software (Dobin et al., 2013). HTSeq-count script (Anders et al., 2015) was used to count the total number of reads matching genic regions. Finally expression value was calculated as RPKM (reads per kilobase per million mapped reads) using the TMM algorithm in EdgeR software (Robinson et al., 2010). The expression level of a *TIMP* gene was represented by an average RPKM of three biological replicates. A *TIMP* gene was defined as foot specific if the following conditions were satisfied: foot RPKM >50 and foot RPKM/non-foot RPKM >100.

### Rapid Amplification of cDNA Ends

In order to obtain the full-length sequences, 5′-RACE and 3′-RACE were carried out, and the primers were designed based on the known nucleic sequence of *Sbp8-1*(CF9441.24) as shown the primer 1 in **Table 1**. The forward primer of 5′-RACE and the backward primer of 3′-RACE are both used the UPM (Universal Primer Mix) from the kit. The 5′-cDNA and 3′-cDNA libraries were constructed using the SMARTer^TM^ RACE cDNA Amplification Kit (Clontech, CA). The resulting products were then cloned into PMD18-T vector to get the PMD18-T-Sbp8-1 plasmid, which was transformed into *E. coli* Top10. Finally, the positive clones were confirmed by sequencing.

**Table 1:** Primers used in this study.

Primer name	Forward(5′-3′)	Backward(5′-3′)
Primer 1	CAAGCGATATTGTCATCATTGGGAAAA	AGTGATGCCACAAACACCTTGGGAGT
Primer 2	CGGGATCCTGTACATGTAATCCATACCCAG	CCGCTCGAGTCAACAATCGTTGTCGGTC
M-C164S	CAGTAATTGAGTCTTTCAACAAAGAACGTTGCGTACCC	CGCAACGTTCTTTGTTGAAAGACTCAATTACTGTTGAATTATCG
M-C186S	GTGGACCGACAACGATTCTTGACTCGAGCACCAC	GTGGTGCTCGAGTCAAGAATCGTTGTCGGTCCAC


### Plasmid Construction and Recombinant Protein Expression and Purification

The *Sbp8-1* gene with termination codon and cleavage sites of restriction endonucleases (*Bam*HI, *Xho*I) were obtained by PCR amplification from the plasmid PMD18-T-Sbp8-1 using primer 2 shown in **Table 1**. The expression vector pET32a was utilized to construct recombinant plasmid, which was transformed into *E. coli* BL21(DE3) for expression. The cultures were grown in LB media with 0.1 mg⋅ml^-1^Ampicillin at 37°C. When the OD_600_ of the cultures reached 0.6–0.8, the cells were induced by adding isopropyl-β-D-thiogalactopyranoside (IPTG) to a final concentration of 0.2 mM. Then, the cultures were grown at 16°C for 15 h. The cells were harvested by centrifuging 3,000 × *g* for 30 min at 4°C and the cell pellets were resuspended in 20 mL ice-cold binding buffer (20 mM Tris–HCl, 20 mM imidazole, 500 mM NaCl, pH 8.5). Cells were lysed by using a sonicator with 5 s pause every 10 s at 40% power for 25 min. The resulting sample was subsequently centrifuged (12,000 × *g*, 25 min, 4°C) to remove insoluble fractions.

The final supernatant was applied to pre-equilibrated Ni-NTA matrix and shaking gently at 4°C for 1 h. After washing with the binding buffer, Sbp8-1 protein was eluted using elution buffer containing 500 mM imidazole in binding buffer. The corresponding fractions were concentrated to 10 mg⋅ml^-1^ for further analysis. The resulting protein was loaded on HiPrep Sephacryl S-200 HR (GE) to monitor the protein polymerization, which was pre-equilibrated with the binding buffer without imidazole. The flow rate is 0.5 ml⋅min^-1^ and the peak fraction was collected and detected by SDS–PAGE. For the polyclonal antibody preparation, the fusion Trx tag of recombinant Sbp8-1 was removed by protease (Supplementary Figure 1).

#### Site-Directed Mutagenesis

QuikChange Site-Directed Mutagenesis Kit (Agilent technology, Santa Clara, CA, United States) was used to perform the site-directed mutagenesis. The primers M-C164S and M-C186S (**Table 1**) were used for the mutation of Sbp8-1^C164S,C186S^. The expression and purification method of the mutant protein is the same as that of Sbp8-1.

### Localization of Sbp8-1 in *Chlamys farreri* Byssus Based on Western Blot

#### Scallop Byssal Protein Extraction

Protein extraction was carried out according to the previous experimental method (Miao et al., 2015). The collected byssus from *Chlamys farreri* were rinsed several times with deionized water and cut into different parts as the picture showed (**Figure 3A**). Briefly, the corresponding fractions were grinded into powder in liquid nitrogen and incubated with extraction buffer (5% acetic (*v/v*), 6 M GdnHCl, 2 mM EDTA, 10 mM DTT) at 37°C for 1 h. After centrifugation (12,000 × *g*, 25 min, 4°C), supernatant was collected and dialyzed against 1% acetic acid (*v/v*) at 4°C. Finally, the extracted protein was dialyzed against deionized water and lyophilized.

#### Preparation of Polyclonal Antibody

The recombinant Sbp8-1 protein was excised from SDS–PAGE gel (Supplementary Figure 1) and ground thoroughly as the antigen. The samples were prepared using a typical procedure (Plumari et al., 2010). After four times immunization, the rabbits were sacrificed and serum was collected for antibody purification (ABclonal Biotechnology, Wuhan, China).

*Western blot:* Western blot analysis of the byssus protein extractions was performed. After electrophoresis, the proteins were electrotransferred to a PVDF membrane for 45 min at 100 V, and the membrane was blocked by incubating the membrane with 5% non-fat milk in Tris-buffered saline containing 0.1% Tween-20 (TBST) for 2 h. The membrane was then incubated overnight at 4°C with rabbit polyclonal anti-Sbp8-1 (1:500 dilution) in blocking solution. After rinsing with TBST, the membrane was incubated with diluted (1:5000) peroxidase-conjugated goat anti-rabbit IgG antibody (BBI, New York, United States) for 1 h. Finally, the membrane blots were developed using an enhanced chemiluminescence regent kit (BOSTER Biological Technology, Wuhan, China).

### Peptide Mass Fingerprinting Derived From Gel Bands

The corresponding band was cut with a clean blade and subjected to trypsin digestion after destaining. After washing with 100 mM NH_4_HCO_3_, the gel pieces were rehydrated in 100 mM NH_4_HCO_3_ with 10 ng sequencing grade-modified trypsin (Promega, WI) at 37°C overnight. After digestion, the protein peptides were collected, and the gels were washed with 0.1% TFA in 60% ACN to collect the remaining peptides (Shevchenko et al., 2006). Then the samples were analyzed using Easy-nLC nano-flow HPLC system connected to Orbitrap Elite mass spectrometer (Thermo Fisher Scientific, CA, United States). Then the raw files were analyzed using the Proteome Discoverer 1.4 software (Thermo Fisher Scientific, CA, United States). The deduced Sbp8-1 sequence was used as the search template.

### Detection of Metalloproteinase Inhibitory Activity

Succinylated gelatin and type IV collagenase (Sigma-Aldrich) were used for the metalloproteinase inhibitor activity test. The gelatin was succinylated using the method described previously (Rao et al., 1997). And the inhibition assay was carried out in the reaction system including 0.14 μM type IV collagenase and 240 μg succinylated gelatin in the presence or absent of Sbp8-1, the total volume was adjusted to 150 μL using 50 mM borate buffer. The reaction buffer was incubated at 37°C for 30 min, then reacted with 0.03% TNBSA and read at 450 nm after incubation at room temperature for 20 min and the relative enzyme activity was monitored.

### Circular Dichroism Spectrum Detection

In order to verify whether the Sbp8-1 protein was folded correctly, the secondary structure of Sbp8-1 was detected by circular dichroism spectrum. The Sbp8-1 protein was digested with protease to remove the fusion tag. After purification, the resulting Sbp8-1 without Trx was dialyzed against phosphate buffer (0.01 M, pH 8.0 with 250 mM Na_2_SO_4_). The protein sample was injected into the quartz cell and then measured within the wavelengths ranging from 190 to 250 nm (detection step of 1 nm and a detection time interval of 0.5 s). During the test, the phosphate buffer was used as a control and the experiment was repeated three times.

### Amino Acid Composition, Sequence Alignment and Structural Modeling

The TIMP-2 protein sequences with the highest structural similarity to the Sbp8-1 protein were selected for amino acid sequence alignment. The alignment was generated with ClustalW (**Figure 4**). The molecular weights and theoretical isoelectric points were analyzed on the Expasy server^2^. Signal peptide was predicted with SignalP 4.1 server^3^.

## Results and Discussions

### Evolutionary Analysis of *TIMP* Genes

To gain insights into the evolution of *TIMP* gene and its expression in different tissues of scallops, we conducted genome-wide search and identified thirteen *TIMP* genes in the *C. farreri* genome. In addition, thirteen *TIMP* genes were identified in scallop *Patinopecten yessoensis* and *oyster Crassostrea gigas* respectively, but none in other molluscs (*Lottia gigantea*, *Octopus bimaculoides*, and *Aplysia californica*). The number of TIMPs in bivalves is remarkably higher than those in other molluscs and non-molluscs (e.g., four TIMPs in human and one in fly) suggesting that *TIMP* gene family in bivalves had expanded notably during evolution.

**FIGURE 1:**
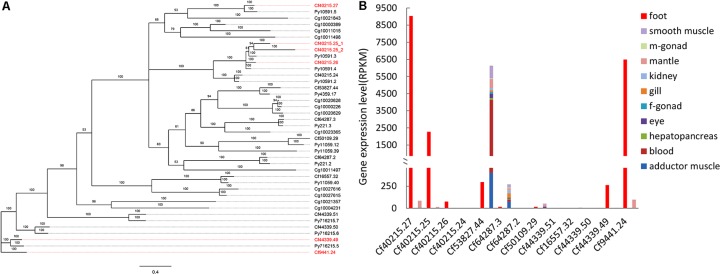
Phylogenetic and expression analysis of *TIMP* genes. **(A)** Bayesian phylogenetic tree of *TIMP* genes in bivalves. Red gene names indicated the foot-specific TIMPs from *C. farreri*. CF: *Chlamy farreri*; PY: *Patinopecten yessoensis*; Cg: *Crassostrea gigas*. **(B)** Expression comparison of *C. farreri TIMP* genes between foot and a sum of other tissues (smooth muscle, male gonad, mantle, kidney, gill, female gonad, eye, hepatopancreas, blood, and adductor muscle).

**FIGURE 2:**
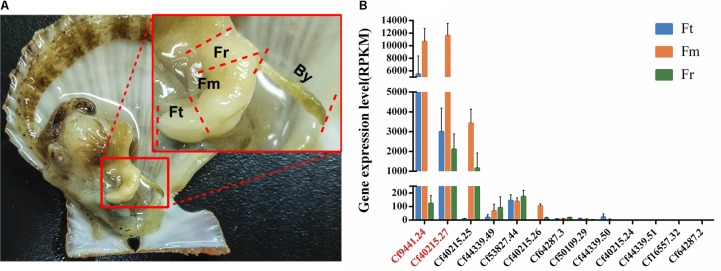
Anatomical structure of *Chlamys farreri* foot and expression analysis of all of *TIMP* genes. **(A)** Anatomy of *C. farreri* and magnified display of *C. farreri* foot. It demonstrates the foot contains three different regions as labeled: Fr, root region of foot; Fm, middle region of foot; Ft, tip region foot; By, *C. farreri* byssus. **(B)** Expression levels of *C. farreri TIMP* genes across different foot regions. The genes labeled in red font encode Sbp8-1 (Cf9441.24) and Sbp7 (Cf40215.27) respectively, the standard errors were based on three biological replicates. This analysis indicates that the genes encoding Sbp8-1 and Sbp7 proteins were highly expressed in the middle region and tip region of *C. farreri* foot.

Our previous study identified seven byssal proteins that were foot specific (Miao et al., 2015). Among them, two *TIMP*s (Sbp7, Sbp8-1) were discovered through our sequence alignment analysis. Phylogenetic analysis (**Figure 1A**) indicated the high diversity and deep evolutionary divergence of *TIMP*s in the bivalve lineage. Many clades contained *TIMP* genes from both scallops and oyster, implying that the duplication events of *TIMP* genes even occurred before the divergence of scallop and oyster lineages (∼425 million years ago) (Wang et al., 2017). The expression analysis of *TIMP*s in *C. farreri* (**Figure 1B**) revealed a substantial number of foot-specific *TIMP*s including Sbp7 and Sbp8-1, with remarkably higher expression levels in foot (RPKM: 70-9,000) than in other tissues (0.3–92.0; **Figure 1B**). These foot-specific *TIMP*s showed the dispersed distribution across the phylogenetic tree, suggesting that their potential functional roles in foot may have an ancient evolutionary origin. Analysis of the expression level of *Sbp8-1* (Cf9441.24) suggests that it is mainly secreted by the middle region and tip region of scallop foot (**Figure 2**). According to previous reports about the distribution of glands in the scallop foot (Gruffydd, 1978), Sbp8-1 protein may be secreted by the second type of mucous cell which is found all along the ventral surface of the middle region and tip region of the foot. Previous studies showed that different types of proteinase inhibitors were identified in the *Zebra mussel* (Xu and Faisal, 2008) and *Mytilus coruscus* byssus (Qin et al., 2016). Also, metalloproteinase inhibitor was identified in the soluble fraction from proximal thread (Qin et al., 2016). Collectively, these findings suggest that TIMP genes may play important roles for the assembly and function of byssus in bivalves, which is likely of ancient evolutionary origin.

### Distribution of Sbp8-1 in *C. farreri* Byssus

**FIGURE 3:**
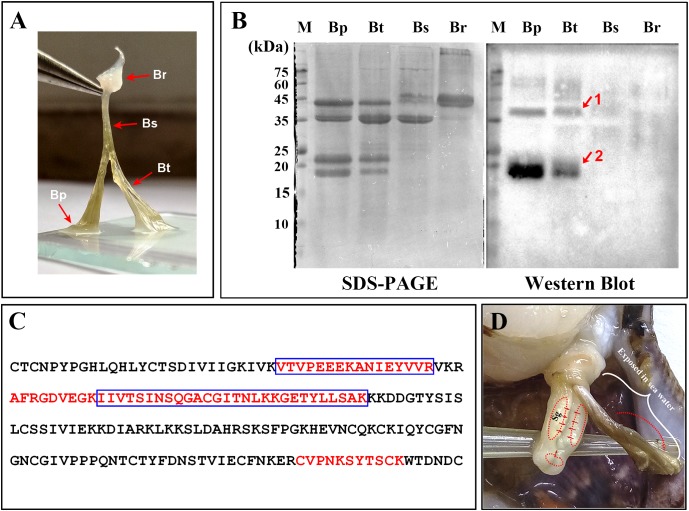
Distribution of Sbp8-1 in *Chlamys farreri* byssus based on western blot. **(A)** Picture of *C. farreri* byssus showing it contains four regions; Br, byssal root region; Bs, byssal sheathed region; Bt, byssal thread region; Bp: byssal plaque region. **(B)** SDS–PAGE and western blot analysis of extracted byssal proteins. The red arrows and numbers indicate the corresponding bands for the further mass spectrometry analysis. **(C)** The identified unique peptide from the corresponding protein bands in **(B)**. The amino acids in the blue box are the peptides identified in Band 1 and the amino acids in the red font are the peptides identified in Band 2. **(D)** The secretion and distribution areas of Sbp8-1. Sg, secretory glands. The red solid arrow indicates the secretion areas of the Sbp8-1 in scallop foot based on the expression level analysis. The red dashed arrow indicates the distribution area of Sbp8-1 in scallop byssus based on the western blot analysis.

Detailed visualization demonstrates that the scallop byssus could be divided into four parts (**Figure 3A**) and the protein was extracted for these individual parts. The SDS–PAGE showed that similar protein compositions were observed for the byssal thread and plaque region (**Figure 3B**). They both contain four major bands with similar Mw (2 bands with Mw around 40 kDa and 2 bands with Mw around 20 kDa). And only one major band was observed with the Mw around 40 kDa for the sheathed region. However, the two major bands (1 band with Mw around 45 kDa and the other one with Mw >75 kDa) were observed for the root region. Similar to our previous observation, the overall scallop byssal thread protein was composed by three major fractions based on the Mw distribution (Miao et al., 2015). In sum, these observations indicate that protein composition vary in different parts of byssus.

To accurately map the distribution of individual compositions in byssus, the western-blot was carried out. Thereby, we first attempted to over-express these two proteins (Sbp7, Sbp8-1) to obtain as the antigen. However, poor expression level of Sbp7 precluded further investigation of this protein, and then the Sbp8-1 was chosen for the following assay. As shown in **Figure 3B**, the western-blot assay using the anti-Sbp8-1 antibody showed Sbp8-1 was discovered mainly in the byssal thread and plaque region, not in the others. However, two bands were observed for both lanes, one band (band 1) is around the 20 kDa, which is close to the Mw of Sbp8-1. Whereas the other band (band 2) is around the 40 kDa, which is one fold higher than the Mw of Sbp8-1. To further validate presence of Sbp8-1 in these two bands, the mass-spectroscopy was carried out. And the result demonstrates that 6 unique peptides were discovered for band 2 and 8 unique peptides for band 1 (**Figure 3C**). This further confirms that the Sbp8-1 was discovered mainly in the byssal thread and plaque region.

Taken together, it was found that the Sbp8-1 was mainly secreted in the scallop foot middle and tip region based on the expression pattern analysis (**Figure 2**), and finally distributed in the byssal thread and plaque region as depicted in **Figure 3D**. However, the transport pathway needs more investigation.

### Amino Acid Composition and Sequence Alignment Analysis

#### Amino Acid Composition Analysis

**FIGURE 4:**
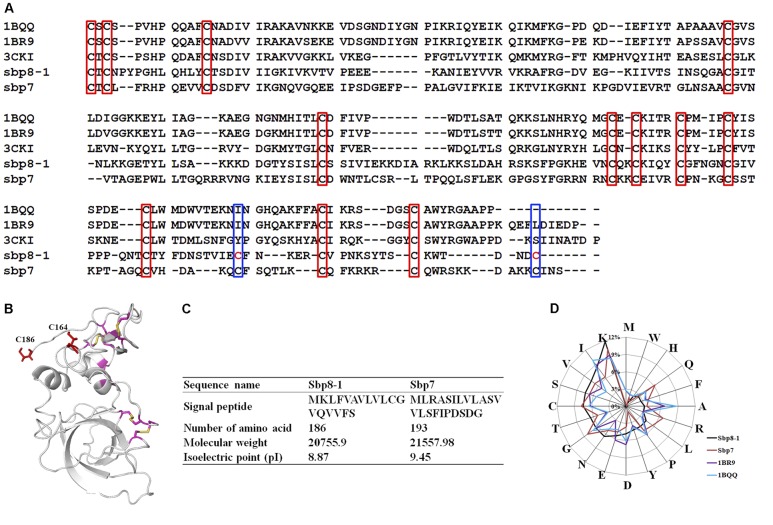
Sequence Characteristics and the basic information of Sbp8-1 and Sbp7. **(A)** Multiple sequence alignments of the Sbp8-1 and Sbp7 with other structure determined TIMPs. Three others structure-determined TIMPs were chosen due to the relative high sequence similarity to either Sbp8-1 or Sbp7. They include the PDB: 1BR9, human TIMP-2; PDB: 1BQQ, bovine TIMP-2; PDB: 3CKI: human TIMP-3). Red boxes indicate the 12 conserved Cys residues and the blue boxes indicate two extra residues in the C-terminal of Sbp8-1. **(B)** The three-dimensional structural model of Sbp8-1 generated by I-Tasser (Yang et al., 2015) suggests that two extra Cys present in Sbp8-1 C-terminal do not form disulfide bonds. **(C,D)** Amino acid composition analysis of Sbp8-1 and Sbp7.

The amino acid analysis for Sbp8-1, Sbp7 was summarized in **Figures 4C,D**. One significant feature for Sbp8-1 and Sbp7 compared to others structure-determined TIMPs is the relative high Lys content, which leads to their basic theoretical pI as shown in **Figure 4C**.

#### Sequence Alignment

Sequence alignment was performed as shown in **Figure 4A** for the two TIMPs from scallop byssal protein (Sbp8-1, Sbp7) with three structure-determined TIMPs (PDB:1BR9, human TIMP-2; PDB: 1BQQ, bovine TIMP-2; PDB:3CKI, human TIMP-3). First, it showed the low sequence similarity between the scallop byssal protein (Sbp8-1, Sbp7) and the other TIMPs (Sequence identity <30%); Furthermore, it was found that the C-terminal region is more divergent, and this might be functional relevant because the C-terminal of TIMP may enhance the inhibitory selectivity and binding efficiencies (Willenbrock et al., 1993). TIMP-2 binds to the hemopexin domain of proMMP-2 to form a tight complex through its C-terminal domain (Visse and Nagase, 2003). More surprising, two additional Cys residues are discovered (blue box in **Figure 4A**). Generally, the TIMPs have 6 disulfides formed by 12 conserved Cys residues (red box in **Figure 4A**). Previous study demonstrated that insertion of Cys residue in C-terminal of human TIMP-3 results in the human Sorsby’s fundus dystrophy (SFD) disease. And the plausible molecular mechanism is the Cys insertion leads to the formation of intermolecular disulfide bridges that cause protein polymerization (Stohr and Anand-Apte, 2012). Also, the structural model of Sbp8-1 generated using the I-Tasser server (Yang et al., 2015) suggests that two extra Cys residues (C164, C186) in Sbp8-1 C-terminal exist as free state (**Figure 4B**). In sum, the above analyses indicate the Sbp8-1 is an atypical TIMP, which might have distinct biochemical features and unique function in scallop byssus.

### Biochemical Characterization of Recombinant Sbp8-1

Biochemical characterization demonstrates that Sbp8-1 is an atypical TIMP, which may function as the cross-linker in the scallop byssus as mentioned below.

#### Polymerization

**FIGURE 5:**
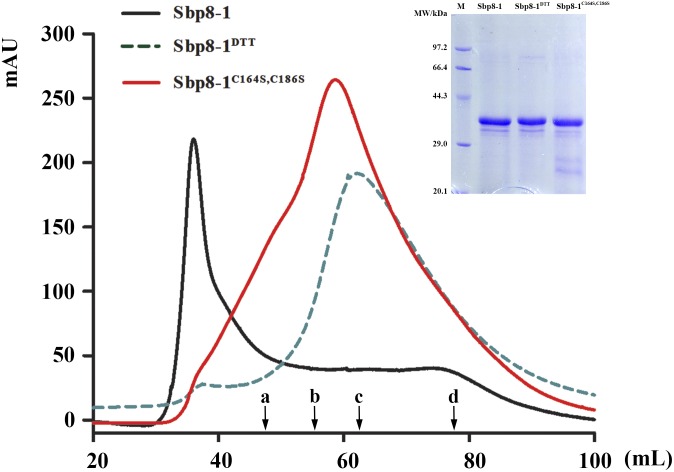
Protein polymerization analysis based on gel filtration. The Sephacryl S-200 High Resolution column was used to evaluate the polymerization state. The solid black line represented the gel-filtration profile of Sbp8-1 running in the binding buffer and the dashed black line represented gel filtration profile of Sbp8-1 running with binding buffer with 10 mM DTT, while the solid red line represented the gel-filtration profile of Sbp8-1^C164S,C186S^. The inset SDS–PAGE shows the purity of each sample. The black arrows indicate the elution volume at the peak of different molecular weight proteins. a, IgG (160 kDa); b, BSA (67 kDa); c, β-lactoglobulin (35 kDa); d, cytochrome C (12.4 kDa).

The recombinant Sbp8-1 (with Trx fusion tag) was loaded on gel filtration chromatography using Hi–Prep Sephacryl S-200 HR (GE) to monitor the polymerization state. As expected, the Sbp8-1 was eluted in void volume (around 36 mL), indicating the polymerization occurs (**Figure 5**). To explore the relationship between the two additional Cys residues (**Figure 3A**) and the polymerization, two following assays were carried out. First, the DTT (10 mM) was added in the elution buffer and it turned out that the elution volume shifted to 60 mL, this suggests the presence of intermolecular disulfides might cause the polymerization, which may formed by the two C-terminal extra Cys residues. To confirm this hypothesis, the C164S and C186S double mutation was then generated. The mutant Sbp8-1^C164S,C186S^ was reloaded on the gel filtration chromatography. The elution volume of Sbp8-1^C164S,C186S^ shifted to the 62 mL as shown in **Figure 5**. Our analyses strongly indicate that Sbp8-1 is in the polymerization state, and this polymerization is caused by the two extra free Cys residues in the C-terminal region. It is known that the free thiol groups are typically important for the marine adhesives. For example, in mussel footprint protein mfp-6 with 11 mol% cysteine content, and most of them occur as free thiol (Lee et al., 2011). It is known that DOPA exists in the scallop byssal thread and plaque region (Li et al., 2017), it is therefore reasonable to propose that Sbp8-1 may function as the cross-linker through the interaction between free cysteine and DOPA.

#### Enzymatic Inhibitory Assay

The biological function annotation based on seqeunce alignment showed that the Sbp8-1 belongs to TIMP-2. Previous study showed that TIMP-2 can inhibit the activity of MMP-2 (type IV collagenase) (Tuuttila et al., 1998). In order to test whether Sbp8-1 protein has inhibitory activity toward type IV collagenase, the succinylated gelatin method was used for the assay. However, it turned out that addition of either Sbp8-1 or the fusion tag control (Trx protein) will enhance the type IV collagenase activity (Supplementary Figure 2), suggests that the Sbp8-1 does not have the inhibitory activity toward type IV collagenase, which is different to the canonical human TIMP-2. To verify the folding of Sbp8-1 protein, the circular dichroism was carried out. The result (Supplementary Figure 3) showed that Sbp8-1 protein has obvious secondary structure. In summary, Sbp8-1 protein is an atypical TIMP without metalloproteinase inhibitor activity.

## Conclusion

**FIGURE 6:**
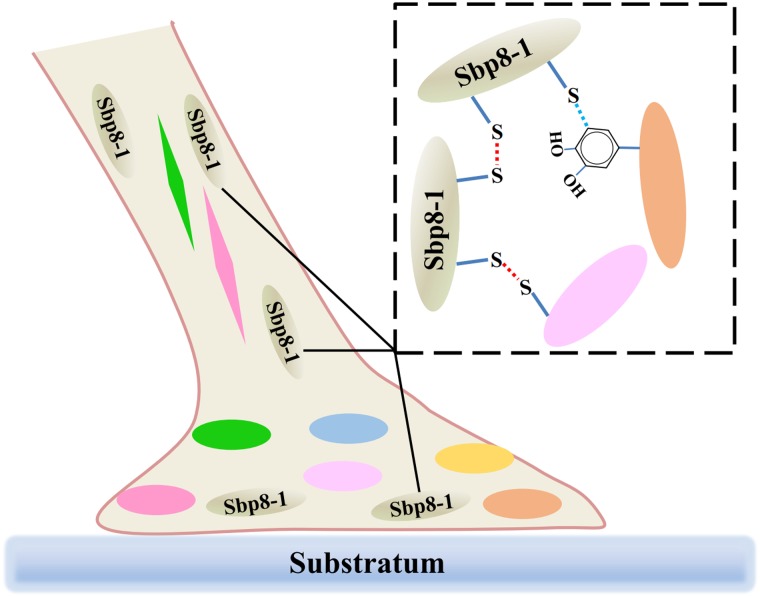
Schematic illustration of the potential cross-linking role of Sbp8-1 protein in the scallop byssus. The left side is a schematic diagram of the structure of the byssus, in which different colors of ovals and diamonds represent different byssal protein components; The dotted box on the right shows the potential cross-linking between Sbp8-1 protein and other proteins.

It is known that both the foot and byssus are crucial for scallop, and the exploration of the scallop byssus composition is the first key step to understand its biological function. Focusing on the TIMPs derived from scallop byssus, a comprehensive characterization of Sbp 8-1 was carried out for the first time. Evolutionary analysis suggests that the *TIMPs* genes of *Chlamys farreri* had gone through multiple gene duplications during evolution, and their potential functional roles in foot may have an ancient evolutionary origin. Focusing on the Sbp8-1, one of the discovered TIMPs from scallop byssus, sequence alignment and enzymatic inhibitory assay suggest that it is an atypical TIMPs. One significant feature is two extra free Cys residues in C-terminal of Sbp8-1. These two residues might form the intermolecular disulfides resulting in the sbp8-1 polymerization; considering its distribution, we proposed that the Sbp8-1 protein may play the cross-linking role in scallop byssus through the C-terminal extra Cys residues as shown in **Figure 6**.

## Author Contributions

WL, QL, SW, and XZ designed and performed the study and drafted the manuscript. SW, ZB, and LW were in charge of reviewing the data analysis during the manuscript submission process. YM, PX, XZ, and PL contributed experiment materials and analysis tools. XD, XZ, and PX collected the information and analyzed the data. BD and SW participated in study development.

## Conflict of Interest Statement

The authors declare that the research was conducted in the absence of any commercial or financial relationships that could be construed as a potential conflict of interest.

## Footnotes

1 https://www.ncbi.nlm.nih.gov/Structure/cdd/wrpsb.cgi

2 https://web.expasy.org/protparam/

3 http://www.cbs.dtu.dk/services/SignalP/
