# Supplementary material for: Characterization of an Atypical Metalloproteinase Inhibitors Like Protein (Sbp8-1) From Scallop Byssus
Source: Front Physiol. 2018 May 23;9:597. doi: 10.3389/fphys.2018.00597 (PMC5975577; doi:10.3389/fphys.2018.00597)
Supplement: Supplementary file 1 [file Data_Sheet_1.pdf]

Characterization of an atypical metalloproteinase inhibitors like protein (Sbp8-1) from scallop byssus

Xiaokang Zhang[1†], Xiaoting Dai[1†], Lulu Wang[1], Yan Miao[1], Pingping Xu[1], Pengyu Liang[1], Bo Dong[1,2,] Zhenmin Bao[1,3], Shi Wang[1,2], Qianqian Lyu[1,2]* and Weizhi Liu[1,2]*

[1]MOE Key Laboratory of Marine Genetics and Breeding, College of Marine Life Sciences, Ocean University of China, Qingdao, China
[2]Laboratory for Marine Biology and Biotechnology, Qingdao National Laboratory for Marine Science and Technology, Qingdao, China
[3]Laboratory for Marine Fisheries Science and Food Production Processes, Qingdao National Laboratory for Marine Science and Technology, Qingdao, China

[†]These authors have contributed equally to this work.

***Correspondence**
Dr. Weizhi Liu
liuweizhi@ouc.edu.cn
Dr. Qianqian Lyu
lqqdo@163.com

Supplemental material

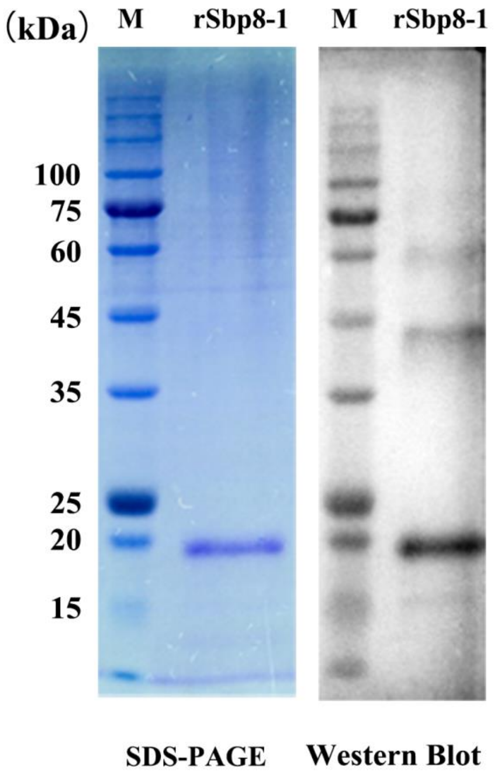

S-Figure 1. SDS-PAGE for the Sbp8-1 protein used as the antigen and western blot of Sbp8-1.

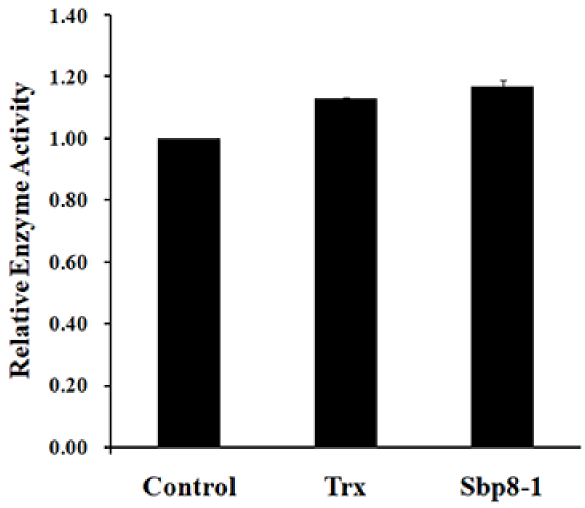

S-Figure 2. Inhibitory activity measurement of Sbp8-1 against type IV collagenase was conducted by succinyl - gelatin method. Each assay was performed in triplicates.

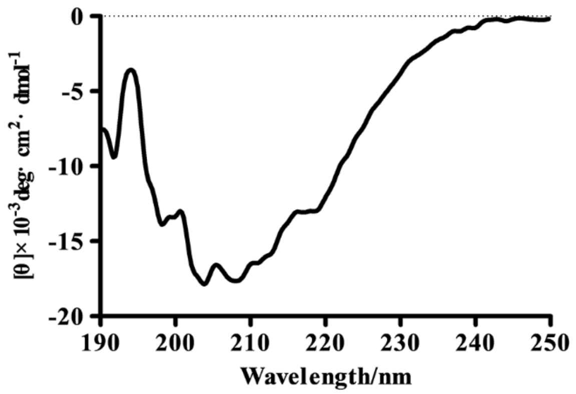

S-Figure 3. Circular dichroism spectrum measurement of Sbp8-1 without Trx tag.
